# High-resolution reanalysis biological ocean data from the Copernicus Marine Service Information for Philippine marine research

**DOI:** 10.1016/j.dib.2025.111913

**Published:** 2025-07-23

**Authors:** Brenna Mei M. Concolis

**Affiliations:** a Department of Climate Extremes and Impacts, Institute of Coastal Systems – Analysis and Modeling, Helmholtz-Zentrum Hereon, Geesthacht 21502, Germany; b Ocean and Climate Laboratory, College of Fisheries and Aquatic Sciences, Mindanao State University – General Santos, General Santos City 9500, Philippines

**Keywords:** Net primary productivity, Zooplankton, Micronekton, Philippines, Reanalysis data, CMEMS

## Abstract

In the absence of observation data, remotely sensed data provides an effective alternative in characterizing spatiotemporal dynamics and patterns of oceanographic data. Some of the most important variables are biomass estimates which describe the productivity of a certain area. Analyzing data with such indices is a useful tool to identify biological hotspots and shifts in concentrations that could be related to phenomenon and changes in the climate. As biomass patterns are crucial in the coastal areas, it is important to utilize data with high resolution at high frequencies (daily) to reduce the bias and capture significant changes in the coast. The E.U. Copernicus Marine Service Information provides reanalysis data of global biomass content that can be freely access by public users. However, problems accessing data could arise for users without prior knowledge of handling large data which is due to the high-resolution properties of the datasets. In addition, processing of large data can be challenging for users with technical hardware limitations. This dataset is provided to help Philippine marine researchers work with net primary productivity, micronekton, and zooplankton, even if they have technical limitations. Daily values, monthly and annual means, climatologies (daily, monthly, and long-term), and anomalies (daily, monthly, and annual) are provided in the public repository. The dataset will allow short-term and long-term analysis in the Philippine waters.

Specifications Table

**Table:** 

Subject	Marine Science, Biological Oceanography
Specific Subject Area	Gridded high-resolution biomass and productivity data in the Philippine seas
Type of Data	Reanalysis data from E.U. Copernicus Marine Service Information (CMEMS)
Variables	Mass content of micronekton Mass content of zooplankton Net primary productivity (NPP)
Data Format	*.csv* and *.rds*
Data Limits	Longitude: 110 – 130°E Latitude: 2 – 30°N Period covered: 01 January 1998 – 31 December 2023
Resolution	Spatial: 0.083° x 0.083° Temporal: daily, monthly, annual
Data Source Location	E.U. Copernicus Marine Service Information [1]
Data Accessibility	Data Identification Number: https://doi.org/10.5281/zenodo.14918590 Direct URL to data: https://zenodo.org/records/14961405
Related Research Article	None

## 1 Value of the Data

• This dataset offers long-term, high-resolution biological reanalysis data for Philippine waters—an ecologically critical region known as the global center of marine biodiversity and one of the top fish-producing nations worldwide. In the absence of extensive in situ observational data, this dataset enables researchers to analyze spatiotemporal patterns and trends in biomass (NPP, zooplankton, and micronekton) from 1998 to 2023.
• The data is optimal for short-term variability assessment (e.g., seasonal productivity shifts) and long-term trend analyses (e.g., climate-related changes) of local NPP, zooplankton, and micronekton estimates in the Philippines from a large dataset over the period 1998–2023.
• By providing a subsetted data restricted to the Philippine boundaries, the dataset significantly reduces the file size, allowing researchers to save memory storage and choose specific local areas of interest only.
• The data provides more flexible file formats (*.csv* and *.rds*) which can be easily used for further analysis by researchers of diverse backgrounds and levels. This is especially helpful for those who are not familiar with ‘netCDF’ file format, which is the format commonly used for storing and compiling large data, used by the Copernicus Marine Service Information. The *.rds* files can be used in R programming language, while .*csv* files can be used and imported in almost all softwares (including geographic information system)–making it accessible even to those with no scientific programming skills.
• The processing and calculation of means, climatologies, and anomalies from multi-year global datasets often require high-performance computing capabilities. This dataset removes that barrier by providing pre-processed products, making it a substantial resource for researchers with limited technical capacities–thus adhering to ‘FAIR’ principles of findability, accessibility, interoperability, and reusability.
• The dataset includes daily, monthly (seasonal), and long-term climatologies which provide reference baseline information for assessing deviations from ‘normal’ conditions. The inclusion of anomalies allows detection of significant events, such as productivity peaks and declines.

## 2 Background

The Philippines, located in the western tropical Pacific, is known as the global center of marine biodiversity. It is home to thousands of marine flora and fauna [[2], [3], [4]] and lies within the Coral Triangle–the global coral reef biodiversity hotspot [4,5]. Additionally, the country has one of the world’s largest fish productions supplying to global markets [6]. In line with this, assessing short- and long- term changes in ocean biological productivity in these waters is crucial.

While the conventional way of estimating productivity in the marine environment uses chlorophyll data [7,8], the emergence of reanalysis data from sources such as the E.U. Copernicus Marine Service Information enables the use of additional biological variables including NPP, zooplankton, and micronekton biomass, for instance [1]. Among these variables, NPP is considered a key proxy parameter in estimating autotrophic production and serves as a baseline for evaluating trophic abundance [9].

The Philippine seas are highly influenced by seasonal monsoons [10], mesoscale eddies, Rossby waves [11], and large-scale external climate forcings [12]. For example, upwellings southwest off Luzon Strait [13] and Zamboanga Peninsula [14] were reported to induce high levels of productivity due to enhance vertical mixing. Moreover, large-scale climate modes such as El Niño Southern Oscillation (ENSO) and Indian Ocean Dipole (IOD) are highly related to NPP changes in the western tropical Pacific, where high positive anomalies coincided with strong El Niño years [12]. These dynamics underscore the region-specific responses of biological variables to oceanographic and climatic drivers [15,16]. Despite these factors, local studies examining spatiotemporal trends and patterns in ocean biological productivity in the Philippines remain limited as opposed to its adjacent seas– South China Sea [10,17,18] and Indonesian waters [16,19,20]. One key limitation may be the availability of high-resolution and accessible long-record data. In the absence of observational data, reanalysis data can provide long historical records of biological data at equally spaced grids and regular temporal frequency–optimal for detecting trends and patterns.

The present paper offers subsetted publicly available reanalysis data of biological variables namely, mass content of micronekton and zooplankton, and NPP in the Philippine seas, and encourages trends and variability studies. In addition, pre-calculated baseline references in the form of daily, seasonal (monthly), and long-term means (also known as ‘climatology’), which are used to quantify how values deviate from the mean or ‘normal’ conditions [21], along with corresponding anomalies are made available.

Furthermore, researchers may explore the effects of physical variables such as sea surface temperature, ocean currents, surface wind, upwelling [18], seasonal monsoons [10,22], and large-scale remote climate forcing backgrounds [19,23] such as Pacific Decadal Oscillation, ENSO [24], and IOD to these biological data to understand the mechanisms that characterize the productivity in the area. Finally, this data can be used as input data for ecological modeling, forecasting, and prediction, which may serve as reference for fisheries and marine resource management and climate change impact assessment.

## 3 Data Description

The data contains data frames or tables with longitude (x), latitude (y), date, and values columns for each biological variable. The files include daily, monthly, and annual values, as well as climatologies and anomalies for 1998–2023. These data are provided in .csv and .rds formats, except for daily values and daily anomalies, which are only in .rds. This is due to the limited capacity of the .*csv* format and the limited memory storage of the repository used to store these data. Nevertheless, users can easily access the *.rds* format in R using the ‘readRDS’ base function and subset the data according to their specific spatial and temporal needs. A sample code will be made available upon request.

## 4 Experimental Design, Materials and Methods

### 4.1 Data source

The raw data was accessed from the Copernicus Marine Service Information using the ‘copernicusmarine’ API toolbox. This toolbox allows fast-track downloading of datasets available in the E.U. Copernicus Marine Data Store, with functions to subset over specific area and time period of interest. To focus on the Philippine local boundaries, data within these geographical coordinates were downloaded: 110 – 130 °E and 2 – 30 °N. These boundaries, however, do not necessarily depict accepted national boundaries. The specific product used was the ‘Global ocean low and mid trophic levels biomass content hindcast’ with the product identifier ‘GLOBAL_MULTIYEAR_BGC_001_033′ (https://doi.org/10.48670/moi-00020) [1]. The spatial resolution is 0.083° x 0.083° with daily fields of various variables. Only three variables were extracted for climatology and anomaly analysis: net primary productivity (‘npp’, mg/m^2^), micronekton biomass (‘mnkc_*epi*’, g/m^2^), and zooplankton biomass (‘zooc’, g/m^2^).

### 4.2 Data processing and calculation

To ensure that pixel-wise mean calculations for all variables were based on sufficiently complete time series, a data quality mask was applied to exclude pixels with excessive missing data. For each pixel, the total number of valid (non-missing) daily values per pixel over the total number of days across the full time period (i.e., 1998–2023) was calculated. This assessment revealed a binary pattern in data availability, with pixels exhibiting either 100 % or 0 % valid observations. Pixels with 100 % valid data corresponded to water bodies, while those with no valid data (0 %) represented land masses–which were then removed prior to means calculations.

The masked data were stored as data frames for easier data manipulation. Daily values were converted to monthly means and annual means. To calculate the daily climatology, means of each day across all years at a given pixel point were calculated such as in Eq. (1):

(1) $$Climatology_{d}=\;\frac{1}{N}\sum_{y=1}^{N}X_{y,d}$$

**Fig. 1:**
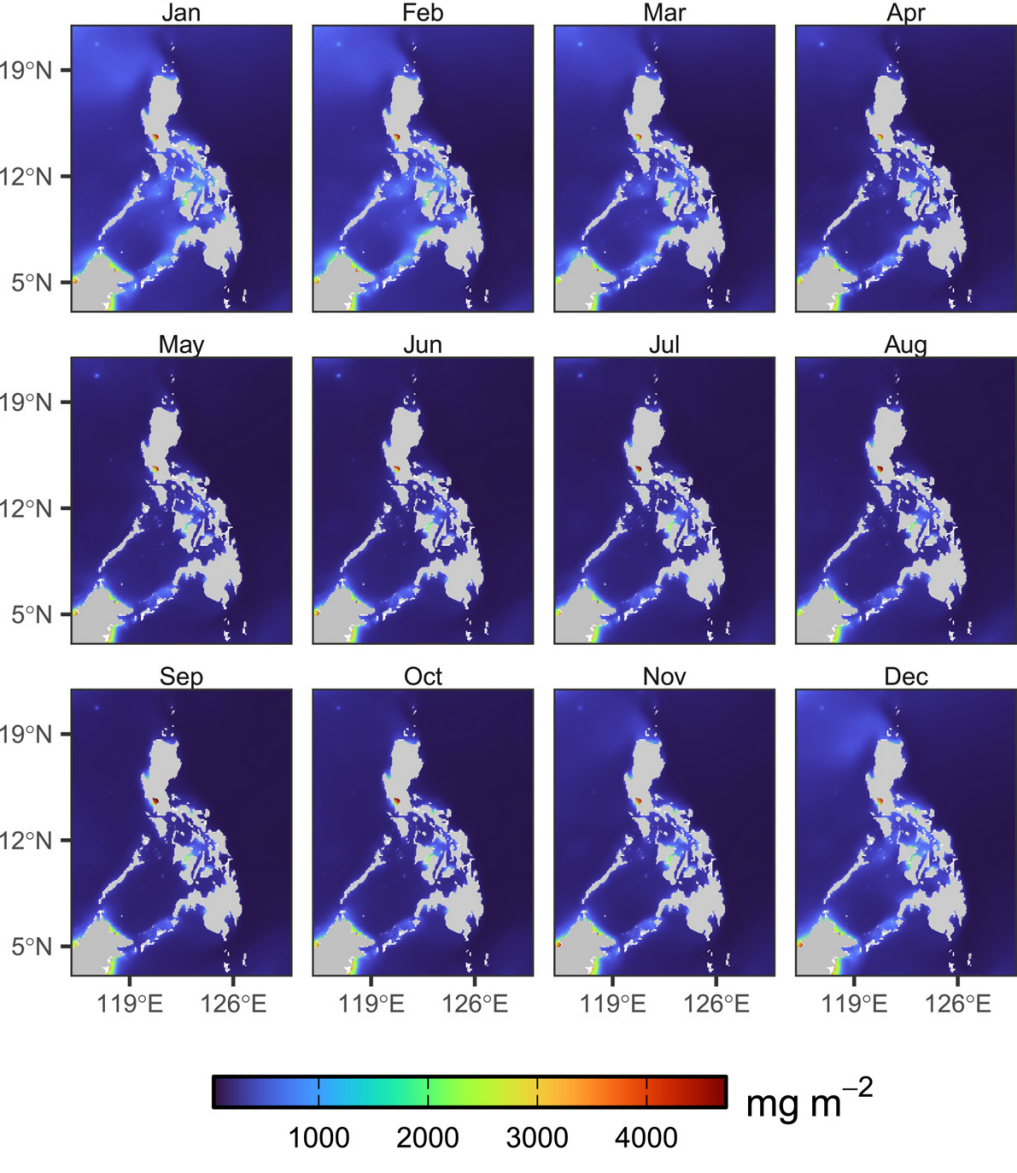
Calculated monthly climatology of NPP across the period 1998–2023.

**Fig. 2:**
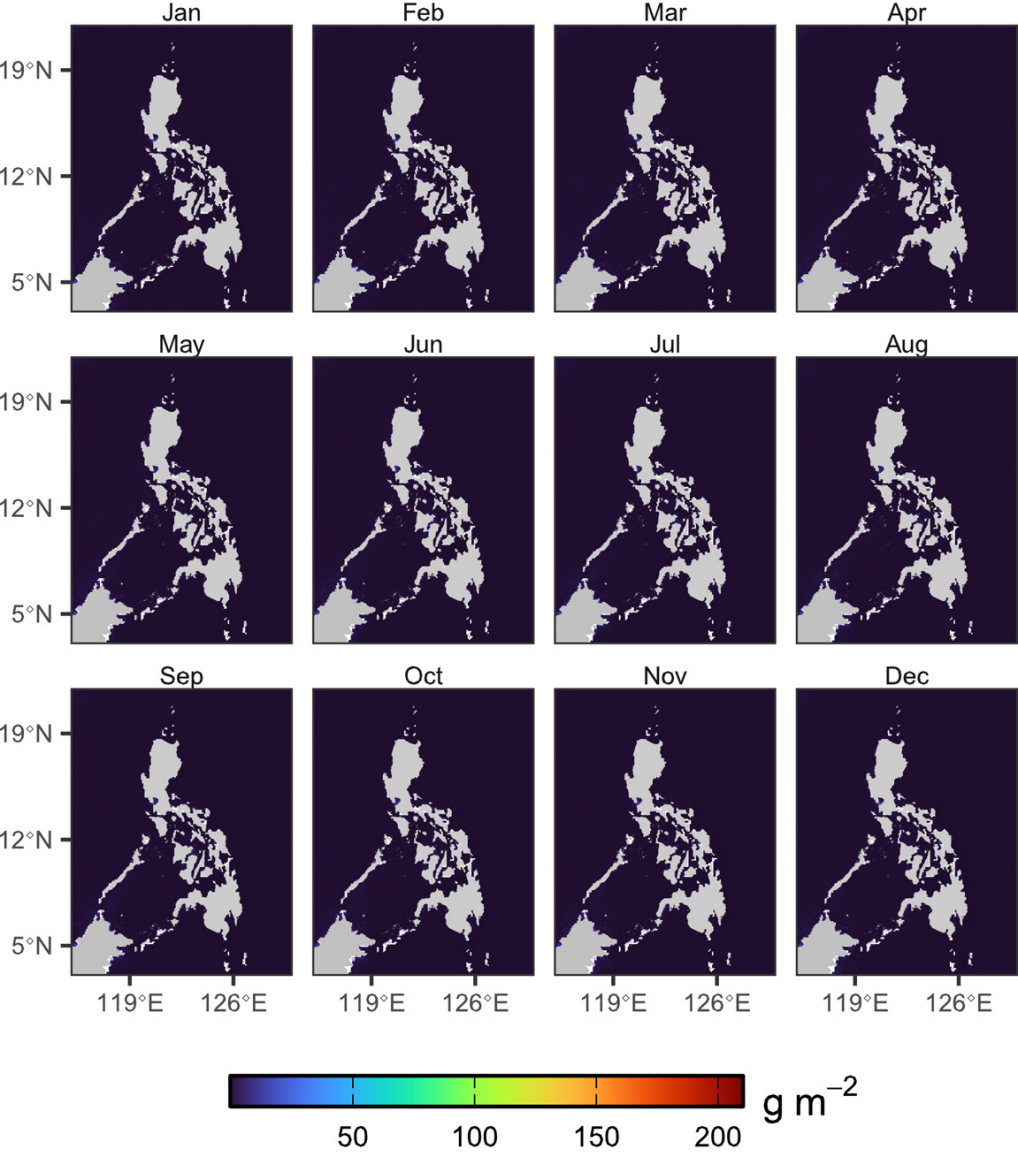
Calculated monthly climatology of micronekton biomass across the period 1998–2023.

**Fig. 3:**
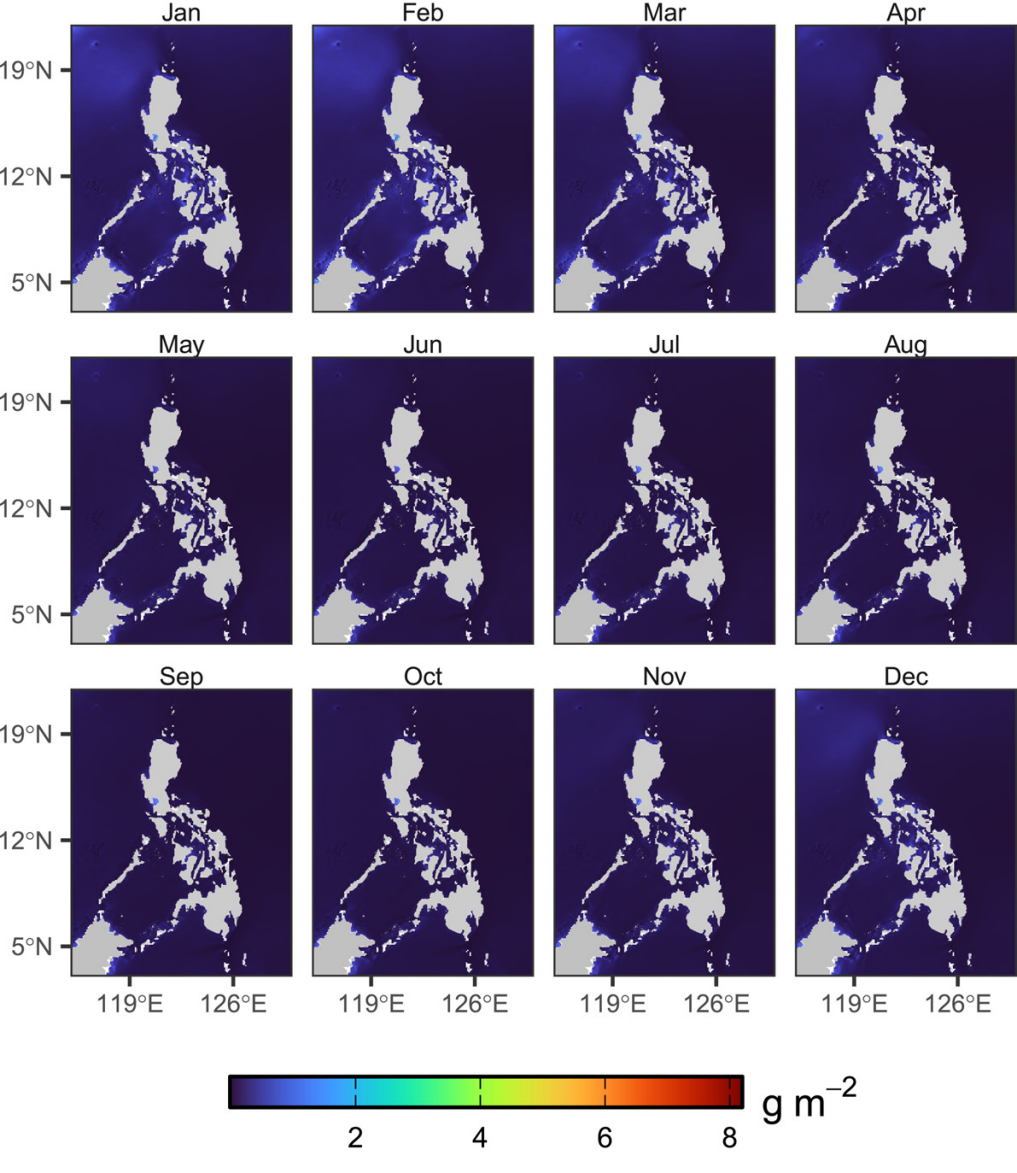
Calculated monthly climatology of zooplankton biomass across the period 1998–2023.

Where: ‘d’ is the day index (1 to 365/365); ‘y’ is the year index; ‘N’ is the number of years (in this case, 26 years), and ‘X’ indicates one of the variables (net primary productivity, zooplankton, or micronekton) values for year ‘y’ and ‘d’. Similarly, monthly climatology, as shown in Fig. 1, Fig. 2, Fig. 3, was calculated by computing for the average of the monthly means across all years at each grid point such as in Eq. (2):

(2) $$Climatology_{m}=\;\frac{1}{N}\sum_{y=1}^{N}X_{y,m}$$

The annual climatology or long-term mean was calculated by averaging the annual means per grid point, which is also referred as the long-term mean/climatology such as in Eq. (3):

(3) $$Climatology_{a}=\;\frac{1}{N}\sum_{y=1}^{N}X_{y}$$

The anomalies were then calculated by removing the climatology from the daily, monthly, and annual actual values per grid cell. All data manipulation and calculation processes were done using the R programming language [25].

## Limitations

‘Not Applicable’

## Data Availability

Reanalysis biological ocean data from CMEMS for the Philippine seas (Original data)

## References

[1] (2024). Global ocean low and mid trophic levels biomass content hindcast, E.U. Copernicus Marine Service Information (CMEMS). Mar. Data Store (MDS).

[2] Pinheiro H.T., Shepherd B., Castillo C., Abesamis R.A., Copus J.M., Pyle R.L., Greene B.D., Coleman R.R., Whitton R.K., Thillainath E., Bucol A.A., Birt M., Catania D., Bell M.V., Rocha L.A. (2019). Deep reef fishes in the world’s epicenter of marine biodiversity. Coral Reefs.

[3] Carpenter K.E., Springer V.G. (2005). The center of the center of marine shore fish biodiversity: the Philippine islands. Env. Biol. Fishes.

[4] VERON J.E.N., DEVANTIER L.M., TURAK E., GREEN A.L., KININMONTH S., STAFFORD-SMITH M., PETERSON N. (2009). Delineating the Coral Triangle, Galaxea. J. Coral Reef Stud..

[5] Asian Development Bank, State of the Coral Triangle: Philippines, 2014. http://coraltriangleinitiative.org/sites/default/files/resources/SCTR-IN.pdf.

[6] TAHİLUDDİN A., TERZİ E. (2021). An overview of fisheries and aquaculture in the Philippines. J. Anatol. Environ. Anim. Sci..

[7] Hu C., Feng L., Lee Z., Franz B.A., Bailey S.W., Werdell P.J., Proctor C.W. (2019). Improving satellite global chlorophyll a data products through algorithm refinement and data recovery. J. Geophys. Res. Oceans.

[8] Yu S., Bai Y., He X., Gong F., Li T. (2023). A new merged dataset of global ocean chlorophyll-a concentration for better trend detection. Front. Mar. Sci..

[9] P.W. Boyd, S. Sundby, H.-O. Pörtner, Cross-chapter box on net primary production in the ocean, climate {change} 2014: {impacts}, {adaptation}, and {vulnerability}. {part} {A}: {global} and {sectoral} {aspects}. {Contribution} of {Working} {Group} {II} to the {Fifth} {Assessment} {Report} of the {Intergovernmental} {Panel} of {Climate} {Change} (2014) 133–136.

[10] Shen S., Leptoukh G.G., Acker J.G., Yu Z., Kempler S.J. (2008). Seasonal variations of chlorophyll a concentration in the northern south China sea. IEEE Geosci. Remote Sens. Lett..

[11] Hu S., Sprintall J., Guan C., Sun B., Wang F., Yang G., Jia F., Wang J., Hu D., Chai F. (2018). Spatiotemporal features of intraseasonal oceanic variability in the Philippine sea from mooring observations and numerical simulations. J. Geophys. Res. Oceans.

[12] Kong F., Dong Q., Xiang K., Yin Z., Li Y., Liu J. (2019). Spatiotemporal variability of remote sensing ocean net primary production and major forcing factors in the tropical eastern indian and Western Pacific ocean. Remote Sens. (Basel).

[13] M. Ecol, P. Ser, D.-L. Tang. 1#, : i-Hsun Ni2, D.R. Ester^, F.E. Muller-Karger4, marine ecology progress series remote sensing observations of winter phytoplankton blooms southwest of the Luzon Strait in the south China sea, (1999).

[14] Villanoy C.L., Cabrera O.C., Yñiguez A., Camoying M., de Guzman A., David L.T., Lament P.F. (2011). Monsoon-driven coastal upwelling off Zamboanga peninsula. Philipp. Oceanogr..

[15] Yun M.S., Kim Y., Jeong Y., Joo H.T., Jo Y.H., Lee C.H., Bae H., Lee D., Bhavya P.S., Kim D., Sun J., Lee S.H. (2020). Weak response of biological productivity and community structure of phytoplankton to mesoscale eddies in the oligotrophic Philippine Sea. J. Geophys. Res. Oceans.

[16] Zhang H.R., Yu Y., Gao Z., Zhang Y., Ma W., Yang D., Yin B., Wang Y. (2023). Seasonal and interannual variability of fronts and their impact on chlorophyll-a in the Indonesian seas. J. Phys. Ocean..

[17] Yu Y., Xing X., Liu H., Yuan Y., Wang Y., Chai F. (2019). The variability of chlorophyll-a and its relationship with dynamic factors in the basin of the south China sea. J. Mar. Syst..

[18] Wei X., Zhao H. (2025). Spatiotemporal distribution of chlorophyll-a concentration in the south China sea and its possible environmental regulation mechanisms. Mar. Env. Res..

[19] Martono M. (2016). Effect of global warming on chlorophyll-a concentration in the Indonesian waters. Makara J. Sci..

[20] Handoko E.Y., Syariz M.A., Hayati N., Putri M., Muryono M., Kuo C.Y. (2024). The spatial–temporal variability of chlorophyll-a across the eastern Indonesian seas region using sentinel-3 OLCI. Appl. Geomat..

[21] Baddour O., Kontongomde H. (2007).

[22] Liu K.K., Chao S.Y., Shaw P.T., Gong G.C., Chen C.C., Tang T.Y. (2002). Monsoon-forced chlorophyll distribution and primary production in the south China sea: observations and a numerical study. Deep Sea Res. 1 Ocean. Res. Pap..

[23] Venegas R.M., Rivas D., Treml E. (2025). Global climate-driven sea surface temperature and chlorophyll dynamics. Mar. Env. Res..

[24] Dang X., Chen X., Bai Y., He X., Arthur Chen C.T., Li T., Pan D., Zhang Z. (2020). Impact of ENSO events on phytoplankton over the Sulu Ridge. Mar. Env. Res..

[25] R.C. Team, R: a language and environment for statistical computing, (2021). https://www.r-project.org/.

